# Device-Derived Digital Biomarkers During SGLT2 Inhibitor Therapy: A Secondary Analysis of the ERASe Trial

**DOI:** 10.3390/jcm15218441

**Published:** 2026-10-29

**Authors:** Ursula Rohrer, Katharina Ingrid Gölly, Martin Benedikt, Viktoria Santner, Martin Manninger, Simon Leiter, Johannes Widkal, Markus Stühlinger, Lukas Herold, Andreas Zirlik, Harald Sourij, Dirk von Lewinski, Daniel Scherr

**Affiliations:** 1 Department of Internal Medicine, Division of Cardiology, Medical University of Graz, Auenbruggerplatz 15, 8036 Graz, Austria; 2 University Clinic of Internal Medicine III/Cardiology and Angiology, Medical University of Innsbruck, Anichstraße 35, 6020 Innsbruck, Austria; 3 Department of Internal Medicine, Division of Endocrinology and Diabetology, Medical University of Graz, Auenbruggerplatz 15, 8036 Graz, Austria; 4 Cardiometabolic Trials Unit, Medical University of Graz, Auenbruggerpl. 2/9, 8036 Graz, Austria

**Keywords:** SGLT2i, digital biomarkers, cardiac implantable electronic devices, heart failure

## Abstract

**Aims:** Implantable cardioverter-defibrillators (ICD) and cardiac resynchronization therapy defibrillators (CRT-D) provide continuous physiological data reflecting heart failure (HF) status and autonomic tone. Sodium–glucose cotransporter-2 inhibitors (SGLT2i) are core HF therapy, but it remains unclear whether their physiological effects are detectable using device-based remote monitoring. Following the ERASe trial, which showed reduced ventricular arrhythmias with ertugliflozin, this post-hoc analysis evaluated its impact on ICD/CRT-D-derived parameters. **Methods:** In this prospective, randomized ERASe post-hoc analysis, ertugliflozin was compared with placebo over 12 months in patients with ICD or CRT-D devices. Device-derived parameters including 24 h heart rate, night heart rate, heart rate variability, physical activity, and thoracic impedance were assessed at baseline and follow-up. Analyses were predefined as exploratory. **Results:** Forty-six patients (22 ertugliflozin, 24 placebo) had ≥1 analysable parameter. Paired longitudinal data were available for 29 patients for 24 h heart rate (HR), 19 for night HR, 17 for HR variability, and 9 for thoracic impedance. No significant between-group differences were observed in 24 h HR, physical activity or thoracic impedance. No significant differential longitudinal treatment effects were observed for the evaluated device-derived parameters, although estimates were imprecise because of limited parameter-specific data availability. Assessment of associations with arrhythmic and heart-failure outcomes was precluded by small event numbers and incomplete parameter-specific data. **Conclusions:** Ertugliflozin did not significantly alter individual device-derived parameters. Larger studies are needed to determine whether device-based physiological signals can identify treatment-related changes.

## 1. Introduction

Heart failure (HF) remains a major global health burden, associated with high morbidity, recurrent hospitalizations and increased mortality despite advances in the foundational medical therapy (FMT) [1]. Importantly, modern cardiac implantable electronic devices collect continuous physiological information beyond arrhythmia detection, offering unique opportunities for remote multiparametric patient monitoring in heart failure patients [2].

Device-based remote monitoring has evolved from single-parameter impedance surveillance to sophisticated multivariable indexes integrating thoracic impedance, heart rate (HR), heart rate variability (HRV), activity level, atrial arrhythmia burden, night HR, respiratory surrogate measures and autonomic tone parameters [2,3,4,5]. Company-specific algorithms have shown promise in HF management [4,6,7,8,9,10], although variability in composition makes comparability difficult [2].

The 2026 ESC Heart Failure Guidelines [1] mention remote monitoring and the 2022 ESC Ventricular Arrhythmia/SCD Guidelines [11] recommend remote monitoring for device integrity surveillance and HF management support. A remote-monitoring-based approach was also suggested to improve GDMT incorporating sodium–glucose cotransporter-2 inhibitors (SLGT2i) [12]. Beyond diagnostics, remote monitoring creates a continuous digital phenotype of the patient, enabling personalized therapeutic strategies and potentially early detection of response or deterioration.

SGLT2i have become a foundational therapy in HF irrespective of glycaemic status, supported by landmark trials such as DAPA-HF, EMPEROR-Reduced, EMPEROR-Preserved, VERTIS, CREDENCE, DECLARE-TIMI 58 and EMMY [13,14,15,16,17,18,19,20,21,22,23]. Their multifaceted pleiotropic benefits—natriuresis, improved ventricular loading, enhanced ketone metabolism, improved endothelial function, reduction in inflammation and fibrosis, autophagy-modifying [24], attenuation of sympathetic drive and restoration of ionic homeostasis—extend beyond glycaemic control, making SGLT2i particularly relevant to arrhythmia-prone HF populations [25,26,27,28,29,30,31]. Emerging evidence suggests SGLT2i may reduce ventricular arrhythmias and appropriate ICD therapies as reported in the main study by Benedikt et al. in the ERASe trial [32,33], yet no prospective study has evaluated whether physiologic device-derived markers reflect treatment response [34,35,36,37].

Remote monitoring could provide a real-time window into cardiorenal metabolic modulation induced by SGLT2i. Improvements in pulmonary congestion surrogates (impedance/HF index), HRV, night HR or physical activity may indicate autonomic stabilization, reduced preload, improved functional capacity or reversal of HF physiology. Small retrospective datasets suggest activity may increase and HR decrease after SGLT2i initiation, while experimental work supports favorable effects on cardiac electrophysiology and substrate remodeling [29,36,38]. Whether such changes translate into quantifiable remote monitoring behavior, or clinical outcomes, in ICD/CRT-D recipients remains unknown.

Given this knowledge gap, we designed this ERASe post-hoc analysis to investigate the effect of ertugliflozin vs. placebo on multiparametric ICD/CRT-D remote monitoring data, congestion trends and physiologic digital biomarkers. Furthermore, we explored whether longitudinal changes in device-derived parameters were associated with subsequent arrhythmic or HF-related clinical outcomes.

## 2. Methods

This study represents a post-hoc analysis of the ERASe trial, the design and results of which have been published previously [32,33,39]. Remote monitoring data were collected at baseline prior to randomization and during treatment with a follow-up of at least 12 months. Remote monitoring data were retrieved from vendor-specific patient management systems and harmonized for analysis.

### 2.1. Informed Consent

All patients signed informed consent. Approval was obtained from the relevant regulatory authorities, including the Ethics Committee of the Medical University of Graz, Austria (EK 32-492 ex 19/20). It was also registered on EudraCT (2020-002581-14) and ClinicalTrials.gov (NCT04600921, first registration 19 October 2020). ERASe was conducted in full conformity with the 1964 Declaration of Helsinki and all subsequent revisions, as well as in accordance with the guidelines laid down by the International Conference on Harmonization for Good Clinical Practice (ICH GCP E6 guidelines).

### 2.2. Study Population

The remote monitoring analysis population comprised all randomized ertugliflozin to reduce arrhythmic burden in ICD/CRT patientS (ERASe) participants with at least one valid device-derived physiological measurement at inclusion and during follow-up, and known treatment allocation (ertugliflozin vs. placebo). The ERASe study, a multicentre, randomized, double-blind, placebo-controlled phase 3b trial, was conducted at Austrian centers between 2021 and 2023. Adult patients with heart failure (LVEF < 50%) despite guideline-directed medical therapy and an implantable cardioverter-defibrillator (ICD) with or without cardiac resynchronization therapy (CRT-D) implanted for >3 months were eligible. Participants were required to have a relevant pre-existing ventricular arrhythmic burden, defined as ≥10 documented non-sustained or sustained ventricular tachycardia episodes within the preceding 12 months, together with at least one additional marker of increased heart failure or arrhythmic risk (NT-proBNP > 500 pg/mL, LVEF < 35%, heart failure hospitalization within the preceding 12 months, >100 non-sustained ventricular tachycardia episodes, or >1 sustained ventricular tachycardia/ventricular fibrillation episode). Additional eligibility criteria included an estimated glomerular filtration rate > 30 mL/min/1.73 m^2^ and haemodynamic stability. Key exclusion criteria comprised ongoing ventricular arrhythmia, pre-existing SGLT2 inhibitor therapy, haemodynamic instability, planned ventricular arrhythmia ablation or device explantation/up- or downgrade, and contraindications to SGLT2 inhibitor therapy. Participants were randomized 1:1 to ertugliflozin 5 mg once daily or matching placebo and followed for 52 weeks. Recruitment was terminated prematurely following changes in heart failure guidelines [40] recommending SGLT2 inhibitor therapy, resulting in a substantially smaller study population than originally planned. Full details of the trial design, eligibility criteria, and primary outcomes have been reported previously [33]. For analyses involving changes over time, only patients with measurements at the corresponding time points were included. No additional exclusion criteria were applied.

Remote monitoring time points included baseline (time point 0; inclusion visit when patients were SGLT2i-naïve) and the 12-month follow-up. If no device interrogation was available at inclusion, the most recent transmission within the preceding 3 months was accepted as baseline. Device data were obtained from vendor-specific remote monitoring systems. Transmissions during hospitalizations or other unplanned follow-up assessments were eligible for inclusion; however, no additional interrogations were available. Individual transmissions were analyzed rather than averaged trend data.

Device-derived physiological parameters were grouped into domains reflecting heart failure status and autonomic regulation. Heart rate metrics comprised mean 24 h heart rate and night heart rate (in patients with <20% atrial pacing). Heart rate variability was assessed using SDNN or vendor-equivalent measures when reliable atrial sensing was available. Physical activity was harmonized across device platforms and categorized into three ordered levels (<1 h/day, 1–3 h/day, and >3 h/day) to enhance cross-platform comparability. Physical activity was analyzed as an ordinal categorical variable and was not treated as a continuous measure. Thoracic impedance was analyzed as a surrogate of pulmonary congestion. Parameter availability varied substantially by device platform and sensing requirements, with detailed completeness rates provided in Supplementary Tables S1 and S2. Congestion indices included vendor-specific multiparametric heart failure metrics (CorVue™ (Abbott, Abbott Park, IL, USA), HeartLogic™ (Boston Scientific, Marlborough, MA, USA), HeartInsight™ (BIOTRONIK, Berlin, Germany), and TriageHF™ (Medtronic, Minneapolis, MN, USA). Because proprietary algorithms and units differ across manufacturers and availability was extremely limited, these indices could not be analyzed quantitatively across the cohort.

### 2.3. Endpoints

Primary treatment-response objective: To evaluate whether device-derived physiological parameters changed differentially over 12 months in patients randomized to ertugliflozin versus placebo.

Secondary exploratory objective: To descriptively report arrhythmic and HF-related clinical outcomes during follow-up.

### 2.4. Handling of Missing Data

Data completeness varied substantially by parameter and time point. Missing device-derived measurements arose through different mechanisms. First, some parameters were structurally unavailable because they were not provided by the respective device manufacturer, device platform, or device generation. Second, availability of individual parameters depended on physiological and sensing requirements; for example, HRV required adequate atrial sensing, and night heart rate was evaluated only in patients with <20% atrial pacing. Thus, missingness reflected a combination of platform-related structural unavailability, measurement-specific eligibility, and unavailable recordings.

Primary analyses were therefore performed using available-case data, with the number of observations reported for each parameter and time point; see Supplementary Tables S1 and S2. Longitudinal analyses were conducted using linear mixed-effects models, which accommodate unbalanced repeated measurements and use all available data under a missing-at-random assumption. Because part of the missingness was structurally related to device capabilities or sensing requirements, the missing-at-random assumption cannot be verified and may not fully hold in the present dataset.

As an exploratory sensitivity analysis, multiple imputation by chained equations was applied to the continuous heart-rate parameters (24 h HR and night HR). Structurally unavailable or non-applicable measurements were excluded from the corresponding imputation population and were not imputed as conventional missing observations. Imputation models included treatment allocation, age, sex, baseline left ventricular ejection fraction and N-terminal pro-brain natriuretic peptide (NT-proBNP). Estimates across imputed datasets were pooled using Rubin’s rules.

Structurally unavailable or non-applicable measurements were excluded from the corresponding imputation population and were not imputed as conventional missing observations.

Analyses involving HRV were restricted to patients with adequate atrial sensing and low atrial pacing and are reported as subgroup analyses. Supplementary Table S2 provides parameter-, visit-, and patient-level availability, illustrating the heterogeneous and partly structural nature of missingness related to device platform capabilities and sensing requirements.

Together, these approaches were intended to maximize use of available device-derived data while acknowledging platform heterogeneity and incomplete transmission patterns.

### 2.5. Statistical Analysis

Continuous variables are presented as mean ± standard deviation or median with interquartile range, as appropriate. Categorical variables are summarized as counts and percentages. Physical activity was treated as an ordinal categorical variable using the predefined categories <1 h/day, 1–3 h/day, and >3 h/day. Baseline comparisons between treatment groups were performed using unpaired *t*-tests or Mann–Whitney U tests for continuous variables and χ^2^ or Fisher’s exact tests for categorical variables.

Longitudinal changes in continuous remote monitoring parameters were analyzed using linear mixed-effects models including fixed effects for time, treatment group, and their interaction. Given the exploratory nature of this post-hoc analysis and the limited sample size, statistical analyses were interpreted descriptively, with emphasis on effect estimates and confidence intervals rather than formal hypothesis testing. A two-sided *p* value < 0.05 was considered nominally significant. As this was a hypothesis-generating post-hoc substudy with multiple exploratory endpoints, no formal adjustment for multiple comparisons was pre-specified.

All analyses were performed using IBM SPSS Statistics version 27 (IBM Corp., Armonk, NY, USA).

### 2.6. Arrhythmic and Clinical Outcomes

Arrhythmic endpoints and clinical outcomes were obtained from the main ERASe dataset. Given the small number of events and limited parameter-specific sample sizes, analyses of device-derived parameters in relation to clinical outcomes were reported descriptively.

## 3. Results

### 3.1. Study Population and Data Linkage

Of the 46 patients analyzed in the main ERASe trial, all 46 participants (22 receiving ertugliflozin, 24 placebo) had at least one analysable device-derived remote monitoring parameter over 12 months FU and were therefore included in this post-hoc analysis.

**Figure 1:**
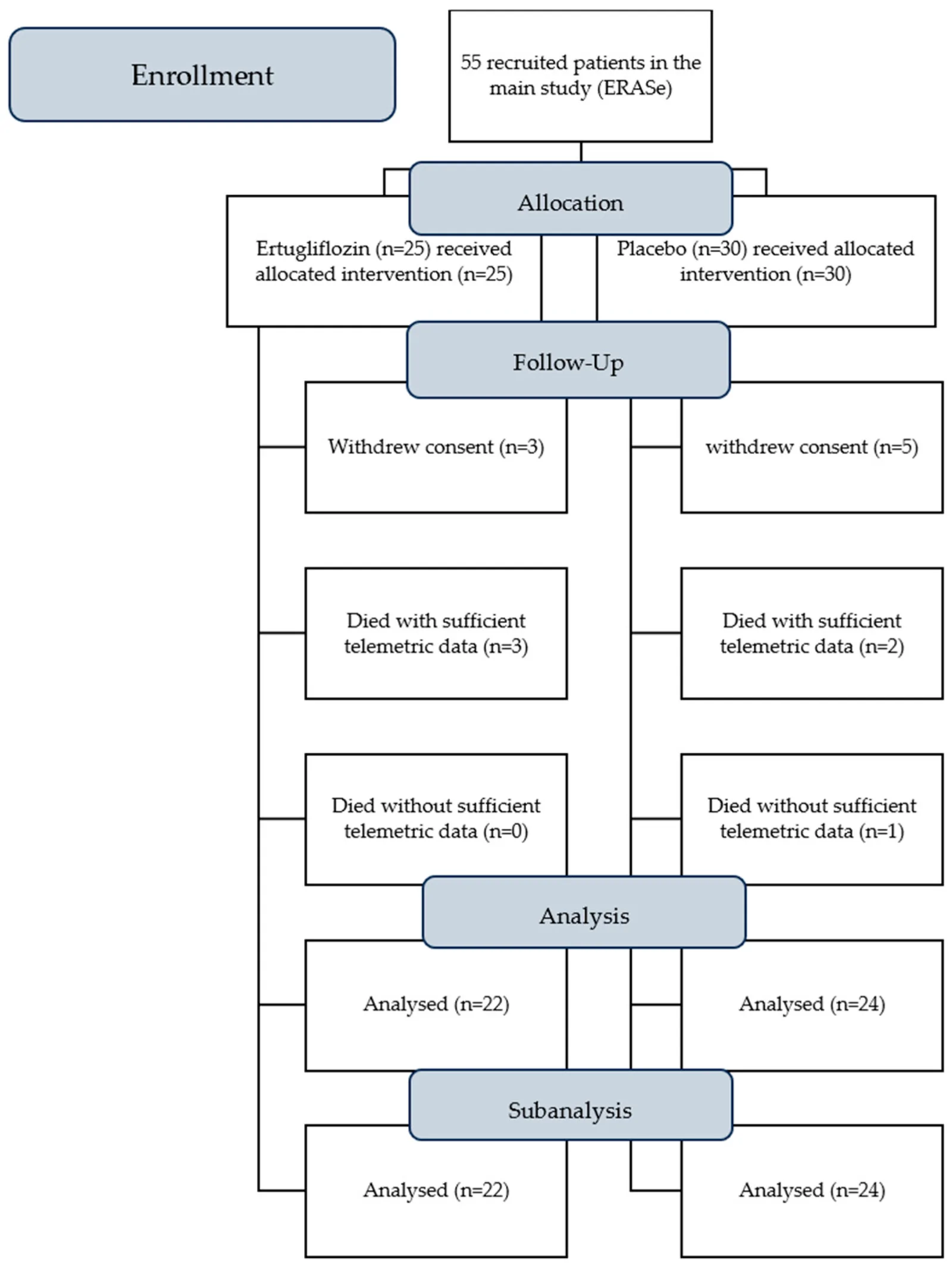
CONSORT Flow chart of post-hoc analysis.

Most patients were implanted with ICD systems (85%), with CRT-D devices in 15% (23 VVI-ICDs, 1 VDD-ICDs, 15 DDD-ICDs, and 7 CRT-D systems). Device configurations and pacing characteristics were comparable between treatment groups, with minimal ventricular pacing in non-CRT systems and near-complete biventricular pacing in CRT recipients. Atrial pacing percentage was 14% in the ertugliflozin group and 16% in the placebo group; ventricular pacing in non-CRT devices was 0% and 1%, and biventricular pacing in CRT devices was 99% and 98% at baseline, respectively. Atrial high-rate episodes were documented in 2 (ertugliflozin) and 4 (placebo) patients with a median burden of 0.2% and 51% (known persistent atrial fibrillation in two patients), respectively; see Figure 1. Due to the low incidence, atrial arrhythmias were not further analyzed. The availability and completeness of device-derived parameters varied across platforms and sensing capabilities; therefore, subsequent analyses are presented according to parameter-specific data availability.

### 3.2. Baseline Remote Monitoring Characteristics

At baseline, the baseline characteristics were comparable between treatment arms (Table 1). Regarding remote monitoring parameters, night HR was higher in the ertugliflozin group compared with placebo (66 ± 11 vs. 58 ± 10 bpm; mean difference 8 bpm, 95% CI 2.3–13.7; *p* = 0.01; Cohen’s d = 0.87). Baseline values for 24 h heart rate (75 ± 10 versus 74 ± 10, *p* = 0.98, Cohen’s d = −0.00), HRV (96 [52; 148] versus 135 [54; 200], *p* = 0.30, Cohen’s d = −0.51) and thoracic impedance (58 ± 10 vs. 61 ± 9 Ω, *p* = 0.76, Cohen’s d = 0.25) were comparable between groups.

During 12-month follow-up, neither 24 h HR nor average daily activity changes were different between groups. The mean change in 24 h HR from baseline to 12 months was +0.4 ± 11 bpm (+1.5 ± 16%) in the placebo cohort vs. +2.7 ± 14 bpm (+4 ± 13%) in ertugliflozin (*p* = 0.59). Daily activity levels and night HR remained relatively stable in most patients, with no significant between-group differences. In the HRV-evaluable subgroup, HRV was 135 [54; 200] at baseline and 120 [39; 178] at 12 months in the placebo group, and 96 [52; 148] and 94 [49; 137], respectively, in the ertugliflozin group. Median change in HRV was −15 [+43; −40] in the placebo group and −1.5 [+6; −50] in the ertugliflozin group. No significant within-group changes were observed (Wilcoxon *p* = 0.301 and *p* = 0.438, respectively), and there were no significant between-group differences at baseline (*p* = 0.30), at 12 months (*p* = 0.359), or in the magnitude of change over time (*p* = 0.228).

However, the observed values, including intrathoracic impedance over time, were small and variable and were derived from a limited number of patients; accordingly, no significant differences between groups were observed (Table 2). Vendor-specific HF indices were not analyzed due to the very limited availability (Supplementary Table S2).

### 3.3. Mixed-Effects Longitudinal Modeling and Multiple-Imputation Sensitivity Analysis

In linear mixed-effects models using all available observations, no treatment × time interaction reached nominal statistical significance. The estimated between-group difference in change was +1.0 bpm for 24 h heart rate (95% CI −6.9 to 9.0; *p* = 0.80), −7.0 bpm for night heart rate (95% CI −14.8 to 0.8; *p* = 0.078), −10.2 ms for HRV (95% CI −51.1 to 30.6; *p* = 0.62), and +14.1 Ω for intrathoracic impedance (95% CI −5.8 to 34.1; *p* = 0.17) (Table 2). Confidence intervals were wide, particularly for HRV and impedance, reflecting the small parameter-specific sample sizes and substantial missingness. Thus, although no statistically significant differential longitudinal treatment effects were identified, several estimates remained imprecise.

Multiple imputation was performed as a sensitivity analysis for 24 h heart rate and night heart rate. Of 92 potential observations per parameter across baseline and 12-month follow-up, 70 (76%) were available for 24 h heart rate and 46 (50%) for night heart rate, corresponding to 22 (24%) and 46 (50%) missing observations, respectively. Following multiple imputation, there was no evidence of a differential change between treatment groups for either parameter. The estimated between-group difference in change over time was −1.0 bpm (95% CI −14.9 to 12.8; *p* = 0.88) for 24 h heart rate and −2.1 bpm (95% CI −17.3 to 13.0; *p* = 0.78) for night heart rate. These estimates were consistent with the overall interpretation of the primary analyses, although uncertainty remained substantial, particularly for night heart rate. Multiple imputation was therefore considered a sensitivity analysis rather than a means of overcoming the limitations associated with substantial and partly structural missingness. Detailed results of the multiple-imputation sensitivity analysis are provided in Supplementary Table S3. Given the baseline imbalance in night heart rate, an additional ANCOVA sensitivity analysis was performed among patients with paired baseline and 12-month measurements (*n* = 19), with 12-month night heart rate as the dependent variable and adjustment for baseline night heart rate. The adjusted between-group difference at 12 months was −5.0 bpm (95% CI −14.2 to 4.1; *p* = 0.26), providing no evidence of a treatment-group difference after adjustment for baseline night heart rate.

### 3.4. Remote Monitoring Parameters and Arrhythmic Outcomes

ICD therapies including ATPs occurred in 15/24 (63%) placebo and 9/22 (41%) ertugliflozin-treated patients, while ICD shocks occurred in 5/24 (21%) and 6/22 (27%), respectively; heart-failure hospitalizations were infrequent. Given the small number of events and substantially reduced parameter-specific sample sizes resulting from incomplete longitudinal device data, no further association or prediction modeling of individual remote-monitoring parameters with clinical outcomes was performed, while previous echocardiographic analyses already suggested no structural changes between the groups [39].

## 4. Discussion

This exploratory ERASe post-hoc analysis is, to our knowledge, the first prospective randomized evaluation of whether treatment with an SGLT2 inhibitor is reflected in device-derived remote multiparametric monitoring signals in ICD/CRT-D recipients. Although ertugliflozin did not produce statistically significant changes across most device-derived parameters compared with placebo, several observations merit consideration within a hypothesis-generating framework.

Baseline remote monitoring parameters were comparable between groups. Over 12 months, ertugliflozin did not significantly affect 24 h heart rate, physical activity, or thoracic impedance, although some parameters showed non-significant directional trends.

Clinical and arrhythmic outcomes were reported descriptively. Given the small number of events and substantially reduced parameter-specific sample sizes, the present dataset was insufficient to meaningfully assess associations between changes in device-derived parameters and subsequent clinical outcomes. Heart-failure hospitalizations were too infrequent for meaningful association modeling. Overall, we did not observe robust between-group differences in longitudinal changes of device-derived physiological parameters. These neutral findings should be interpreted in the context of limited statistical power, substantial missing data, heterogeneity of device platforms and algorithms, and the inherent variability of remote monitoring signals in real-world ICD/CRT-D populations. The study was not powered to detect small-to-moderate changes in continuous physiological measures; effect sizes were modest, confidence intervals wide, and individual trajectories highly variable. Accordingly, the absence of statistical significance does not exclude subtle treatment effects that this analysis was underpowered to detect.

### 4.1. Exploratory Changes in Autonomic Parameters

Neither night heart rate nor HRV demonstrated a statistically significant differential longitudinal response to ertugliflozin, and both findings require cautious interpretation because of substantial data limitations.

Night heart rate was substantially higher in the ertugliflozin group at baseline, raising the possibility that the subsequent numerical reduction was at least partly attributable to regression to the mean. An ANCOVA sensitivity analysis adjusting 12-month night heart rate for its baseline value did not demonstrate a significant between-group difference (adjusted difference −5.0 bpm; 95% CI −14.2 to 4.1; *p* = 0.26). The observed trajectory should therefore not be interpreted as evidence of improved autonomic regulation.

HRV was evaluable longitudinally in only 8 ertugliflozin-treated and 9 placebo-treated patients. There were no significant between-group differences at baseline, at 12 months, or in change over time, and the mixed-effects model yielded an imprecise treatment × time estimate (−10.2 ms; 95% CI −51.1 to 30.6; *p* = 0.62). Consequently, the HRV observations are considered exploratory and do not provide evidence of an effect of ertugliflozin on autonomic modulation.

Although both night heart rate and HRV are physiologically relevant markers of autonomic regulation, the present dataset is insufficient to determine whether SGLT2 inhibition produces measurable changes in these device-derived parameters. Larger studies with systematic and longitudinal parameter acquisition would be required to address this question.

SGLT2 inhibitors have previously been associated with several potential cardiovascular mechanisms, including natriuresis, altered ventricular loading conditions, and modulation of sympathetic activity. However, these mechanisms were not directly assessed in the present substudy, and the device-derived parameters evaluated here cannot establish whether any such mechanisms occurred. Accordingly, the present findings should not be interpreted as mechanistic evidence for an effect of ertugliflozin on autonomic balance, preload, or congestion. Several effects proposed to underline the cardiovascular benefits of SGLT2 inhibition, including changes in volume regulation, myocardial energetics, and autonomic activity, have been described across studies of different SGLT2 inhibitors. These observations provide class-level biological context but should not be interpreted as evidence of ertugliflozin-specific mechanisms. In the present study, ertugliflozin did not produce statistically significant differential changes in the evaluated device-derived physiological parameters, and the study was not designed to establish the mechanisms underlying the cardiovascular effects of ertugliflozin.

Further investigation of device-derived physiological parameters remains warranted because continuous device monitoring represents a potentially useful approach to characterizing treatment-related physiological changes in patients with heart failure. Larger prospective datasets with more complete and harmonized longitudinal measurements will be required to determine whether these parameters can reliably detect such changes.

### 4.2. Context of Current Guideline-Directed Medical Therapy

Interpretation of these findings must also consider the contemporary therapeutic landscape. SGLT2i are now universally recommended as foundational medical therapy for HF across the spectrum of ejection fraction, irrespective of glycaemic status [1]. Consequently, new placebo-controlled randomized trials specifically designed to assess device-derived physiological signals under SGLT2 inhibition are unlikely to be ethically or practically feasible.

This reality highlights the importance of extracting maximal insight from exploratory datasets such as ERASe. Future evidence in this field will likely derive from pragmatic studies, registry-based analyses, within-patient longitudinal comparisons, and dedicated digital biomarker validation efforts rather than traditional randomized controlled trials. In this context, the present post-hoc analysis should be viewed as hypothesis-generating groundwork, defining feasible effect sizes, identifying candidate signals, and clarifying methodological constraints for future investigations.

### 4.3. Methodological Considerations and Limitations

Several limitations merit explicit emphasis. First, data completeness varied substantially across parameters and time points, particularly for HRV and thoracic impedance, limiting inferential robustness. Missingness was partly structural and platform-dependent; therefore, the missing-at-random assumption cannot be fully verified, and results should be interpreted with caution. Multiple imputation should therefore be interpreted as a sensitivity analysis assessing the robustness of the available-case findings rather than as a means of fully overcoming the limitations associated with structurally unavailable device-derived measurements.

Another limitation of this study is that continuous longitudinal device data could not be harmonized across different vendor-specific platforms. Due to substantial variability in data structure and export capabilities, analyses were restricted to predefined discrete time points rather than full trend evaluation. The small HRV-evaluable sample increases the risk of model overfitting, unstable estimates, and inflated type I error, thereby limiting the strength of any subgroup inferences.

Second, vendor heterogeneity affected not only parameter availability but also measurement comparability. Device-derived HRV, physical activity, thoracic impedance, and proprietary multiparametric heart failure indices are not necessarily measurement-equivalent across manufacturers because sensing methods, signal processing, calculation algorithms, reporting intervals, and units differ between platforms. Consequently, nominally similar parameters may capture partly different physiological information across devices, introducing measurement heterogeneity that cannot be fully addressed statistically. This heterogeneity also precluded pooling of manufacturer-specific multiparametric heart failure indices into a unified composite measure. Manufacturer-specific contributions to each parameter and time point are provided in Supplementary Table S4. Given the small and variable parameter-specific sample sizes, manufacturer-restricted analyses would result in very small subgroups and were therefore not considered sufficiently robust to meaningfully address this heterogeneity. Residual manufacturer-related measurement differences must therefore be considered when interpreting the findings.

The premature termination of the ERASe trial warrants specific consideration. Importantly, the decision to discontinue recruitment was not driven by safety concerns, futility, or findings emerging from the trial itself, but by a substantial change in the external clinical landscape. Following the publication of the 2021 ESC heart failure guidelines [40], SGLT2 inhibitors became recommended as part of foundational therapy for heart failure patients. Consequently, continued randomization to placebo became increasingly difficult to justify and recruitment was terminated before the originally planned sample size had been reached.

This illustrates an inherent challenge of investigator-initiated placebo-controlled trials evaluating rapidly evolving pharmacological therapies. Although the study protocol and design were investigator-initiated, conduct of the trial depended on pharmaceutical support, including provision of study medication and placebo, while changes in guideline-directed therapy occurred faster than trial recruitment could be completed. The resulting premature termination substantially reduced the available study population and statistical power. Thus, the small sample size reflects a change in standard of care rather than recruitment failure or a trial-related safety or efficacy signal and should be considered when interpreting the neutral findings of this substudy.

Finally, multiple exploratory analyses were performed without formal adjustment for multiplicity, increasing the possibility of false-positive findings.

Nevertheless, transparent reporting of missing data patterns highlights the limitations of the available dataset. These limitations substantially restrict the inferential strength of the present analysis and emphasize the need for adequately powered prospective studies with systematic and harmonized acquisition of device-derived physiological parameters.

## 5. Conclusions

This exploratory post-hoc analysis did not demonstrate statistically significant differential effects of ertugliflozin on individual ICD/CRT-D-derived physiological parameters. Interpretation was limited by substantial parameter-specific missingness, small effective sample sizes, and vendor-related measurement heterogeneity. Larger studies with systematic and harmonized longitudinal data acquisition are needed to determine whether device-derived physiological parameters can serve as treatment-response markers in heart failure.

## Figures and Tables

**Table 1:** Clinical and device-derived baseline parameters of Ertugliflozin and Placebo group. Data are presented as mean ± SD or *n* (%), as appropriate. ACE-I, angiotensin-converting enzyme inhibitor; ARB, angiotensin receptor blocker; ARNI, angiotensin receptor blocker and neprilysin inhibitor; eGFR, estimated glomerular filtration rate; LVEF, left ventricular ejection fraction; MRA, mineralocorticoid receptor antagonist; NTproBNP, N-terminal pro-brain–type natriuretic peptide; SD, standard deviation.

	Ertugliflozin (*n* = 22)	Placebo (*n* = 24)
Age, years	65 ± 11	66 ± 12
Female sex, *n* (%)	2 (9%)	2 (8%)
BMI, kg/m^2^	28.6 ± 5.3	28.2 ± 6.3
LVEF, %	37 ± 11%	37 ± 8%
NT pro-bnp, pg/mL	1782 ± 2311	2370 ± 2714
eGFR/, mL/min/1.73m^2^	61 ± 18	65 ± 21
ACE-I/ARB or ARNI, *n* (%)	13 (59)	12 (50)
Beta-blocker, *n* (%)	18 (82)	21 (88)
MRA, *n* (%)	12 (55)	14 (58)
Loop diuretic, *n* (%)	9 (41)	15 (63)

**Table 2:** Available case analysis of longitudinal changes of multiparametric monitoring parameters from baseline to 12 months follow-up. Treatment × time estimates were derived from linear mixed-effects models including fixed effects for treatment, time, and their interaction and a patient-level random intercept. Models used all available observations; therefore, model-based estimates may differ from descriptive changes calculated only among patients with paired measurements. HR, heart rate; HRV, heart rate variability.

Parameter	Observations Baseline/12 months	Δ Ertugliflozin	*n* Paired	Δ Placebo	*n* Paired	Treatment × Time Estimate	95% CI	Nominal *p*
24 h HR (bpm)	41/32	+2.7 ± 14	15	+0.4 ± 11	14	+1.0	−6.9 to +9	0.80
Night HR (bpm)	27/22	−0.6 ± 6.9	11	+6.3 ± 11.8 bpm	8	−7.0	−14.8 to +0.8	0.078
HRV (ms)	22/17	−2 [+6; −50]	8	−15 [−40; +43]	9	−10.2	−51.1 to +30.6	0.62
Intrathoracic impedance (Ω)	11/13	0 [+2; −2]	5	−9 [−49; +2]	4	+14.1	−5.8 to +34.1	0.17

## Data Availability

The original contributions presented in this study are included in the article/supplementary material. Further inquiries can be directed to the corresponding author(s).

## Supplementary Materials

The following supporting information can be downloaded at: https://www.mdpi.com/article/10.3390/jcm15218441/s1, Supplementary Table S1: Availability of device-derived remote monitoring parameters across vis-its and treatment groups. Supplementary Table S2: Availability of vendor-specific parameters. Supplementary Table S3: Multiple-imputation sensitivity analysis of longitudinal heart-rate parameters. Supplementary Table S4: Manufacturer-specific availability of device-derived re-mote-monitoring parameters.

## Abbreviations

CRT-D	cardiac resynchronization therapy defibrillator
ESC	European Society of Cardiology
FU	follow-up
GDMT	guideline-directed medical therapy
HF	heart failure
HR	heart rate
HRV	heart rate variability
ICD	implantable cardioverter-defibrillator
LVEF	left ventricular ejection fraction
NT-proBNP	N-terminal pro–B-type natriuretic peptide
SGLT2i	sodium–glucose cotransporter-2 inhibitor
VT	ventricular tachycardia
VF	ventricular fibrillation
