# Supplementary material for: Device-Derived Digital Biomarkers During SGLT2 Inhibitor Therapy: A Secondary Analysis of the ERASe Trial
Source: J Clin Med. 2026 Oct 29;15(21):8441. doi: 10.3390/jcm15218441 (PMC13764287; doi:10.3390/jcm15218441)
Supplement: Supplementary file 1 [file jcm-15-08441-s001.zip › jcm-4534368-supplementary.pdf]

	HR_V1	HR_V2	Night HR_V1	NightHR_V2	HRV_V1	HRV_V2	Impedance_V1	Impedance_V2	Daily Activity_V1	Daily Activity_V2	Optivol_V1 Medtronic	Optivol_V2 Medtronic	Respiratory Rate_V1 Boston Scientific	Respiratory Rate_V2 Boston Scientific	PVCs_V1 Biotronik	PVCs_V2 Biotronik	Sleep Incline_V1 Boston Scientific	Sleep Incline_V2 Boston Scientific	Availability per patient (%)(
Placebo group (n=24)	1	0	0	0	0	0	0	0	1	0	0	0	1	1	0	0	0	0	22
	1	1	0	1	1	1	0	1	0	1	0	1	0	0	0	0	0	0	44
	1	1	0	0	1	1	0	0	1	1	0	0	1	1	0	0	0	0	44
	0	1	0	1	1	1	0	1	1	1	0	1	0	0	0	0	0	0	44
	0	1	1	1	0	0	1	1	0	1	0	0	0	0	0	0	0	0	39
	1	1	0	0	1	1	0	0	1	1	0	0	1	1	0	0	0	0	44
	1	1	0	0	0	0	0	0	1	1	0	0	1	1	0	0	0	0	33
	1	0	0	0	0	0	0	0	1	1	0	0	0	0	0	0	0	0	17
	0	0	0	0	0	0	0	0	1	1	0	0	0	0	0	0	0	0	11
	1	0	1	0	0	0	1	1	1	1	1	0	0	0	0	0	0	0	39
	1	1	0	0	0	0	0	0	1	1	0	0	1	1	0	0	0	0	33
	1	1	1	1	0	0	0	0	1	1	0	0	1	1	0	0	0	0	44
	1	1	1	1	1	1	0	0	1	1	0	0	0	0	0	0	0	0	44
	1	0	1	0	1	1	0	0	1	1	0	0	0	0	0	0	0	0	33
	1	1	1	1	0	0	0	0	1	1	0	0	0	0	1	0	1	0	44
	1	1	1	1	0	0	0	0	1	1	0	0	0	0	0	0	0	0	33
	1	0	1	0	1	0	0	0	1	1	0	0	0	0	0	0	0	0	28
	1	1	1	1	1	0	0	0	1	1	0	0	0	0	0	0	0	0	39
	1	0	0	0	1	1	0	0	0	0	0	0	1	1	0	0	0	0	28
	0	0	0	0	0	0	0	0	1	1	0	0	1	1	0	0	0	0	22
	1	1	1	1	0	0	0	0	1	1	0	0	0	0	0	0	0	0	33
	1	1	1	1	1	1	1	1	1	0	1	1	0	1	0	0	0	1	72
	1	1	1	0	1	1	1	1	0	0	0	0	0	0	0	0	0	0	39
	1	1	1	0	0	0	0	0	0	0	0	0	0	0	0	0	0	0	17
Ertugliflozin group (n=22)	1	1	1	1	1	1	1	1	1	1	0	0	0	0	1	0	1	0	67
	1	1	1	1	1	1	1	1	1	1	0	0	1	1	0	0	0	0	67
	1	0	0	0	0	0	1	0	1	1	0	0	0	0	0	0	0	0	22
	1	1	1	1	1	0	1	1	1	1	1	1	0	0	0	0	0	0	61
	1	0	0	0	1	1	0	0	1	0	0	0	1	1	0	0	0	0	33
	1	1	0	1	1	1	0	1	1	1	0	0	1	1	0	0	0	0	56
	1	1	0	0	1	1	0	0	1	0	0	0	0	0	0	0	0	0	28
	1	1	1	1	1	1	0	0	1	1	1	1	0	0	0	0	0	0	56
	1	0	1	0	0	0	0	0	1	1	1	0	0	0	0	0	0	0	28
	1	0	1	0	1	0	1	0	1	1	1	0	0	0	0	0	0	0	39
	1	1	0	0	0	0	0	0	1	1	0	0	1	1	0	0	0	0	33
	1	1	0	0	0	0	0	0	1	1	0	0	0	0	0	0	0	0	22
	0	1	0	0	0	0	0	0	0	0	0	0	1	1	0	0	0	0	17
	1	1	1	1	0	0	0	0	1	1	0	0	0	0	0	0	0	0	33
	1	1	1	1	0	0	0	0	1	1	0	0	0	0	0	0	0	0	33
	1	0	1	0	1	1	0	0	1	0	0	0	0	0	0	0	0	0	28
	1	1	1	1	1	1	0	0	1	1	0	0	0	0	0	0	0	0	44
	1	1	1	1	0	0	0	0	1	1	0	0	0	0	0	0	0	0	33
	1	1	1	1	0	0	1	1	1	1	0	0	0	0	0	0	0	0	44
	1	0	1	1	0	0	0	0	1	1	0	0	0	0	0	0	0	0	28
	1	1	1	1	0	0	1	1	1	1	0	0	0	0	0	0	0	0	44
	1	1	0	0	1	0	0	1	1	0	1	1	0	0	0	0	0	0	39
Availability per parameter (%)	89	70	59	48	43	37	24	28	87	78	15	13	28	30	4	0	4	2	

Supplementary Table S1: Availability of device-derived remote monitoring parameters across visits and treatment groups. This heatmap illustrating data availability (1 = available, 0 = not available) for device-derived parameters at Visit 1 (V1) and Visit 2 (V2). Rows represent individual patients, grouped by treatment allocation (Placebo and Ertugliflozin). Columns display vendor-independent parameters (24h heart rate [HR], night HR, heart rate variability [HRV], intrathoracic impedance, and daily activity) and vendor-specific parameters (OptiVol for Medtronic devices; respiratory rate and sleep incline for Boston Scientific devices; premature ventricular contractions [PVCs] for Biotronik devices). The rightmost column indicates the percentage of available parameters per patient, while the bottom row shows the overall availability percentage per parameter across the cohort. Vendor-specific parameters are only applicable to

patients implanted with the corresponding device platform. This visualisation highlights the distribution and structure of missing data across parameters, visits, and device platforms.

Vendor-independent parameters (entire cohort)				
**Parameter**	**Baseline availability**		**12-month availability**	
HRV	43%		30%	
Night HR	59%		48%	
Thoracic impedance	24%		28%	
Daily Activity	87%		78%	
**Vendor-specific parameters (availability reflects device platform)**				
	**Baseline availability (percentage relative to vendor-specific subgroups)**	**Baseline availability (percentage relative to total cohort)**	**12-month availability (percentage relative to vendor-specific subgroups)**	**12-month availability (percentage relative to total cohort)**
**Medtronic devices (n = 13)**				
Optivol Index	54% (7/13)	15% (7/46)	46% (6/13)	13% (6/46)
TriageHF™	n.a.	n.a.	n.a.	n.a.
**Boston Scientific (n=19)**				
Respiratory Rate	74% (13/19)	28% (13/46)	74% (14/19)	30% (14/46)
Sleep Incline	11% (2/19)	4% (2/46)	5% (1/19)	2% (1/46)
HeartLogic™	n.a.	n.a.	n.a.	n.a.
**Biotronik (n=2)**				
No of PVCs	100% (2/2)	4% (2/46)	0%	0%
HeartInsight™	n.a.	n.a.	n.a.	n.a.
**Abbott (n=12)**				
CorVue™	n.a.	n.a.	n.a.	n.a.

Supplementary Table S2: Availability of vendor-specific parameters. Percentages are shown relative to the total cohort and, where applicable, relative to the vendor-specific subgroup. Missingness is partly structural due to platform capabilities and sensing requirements.

Parameter	**Available baseline, n/N (%)**	**Available 12 months, n/N (%)**	**Missing/imputed observations, n/N (%)**	**Difference in change, estimate (95% CI)**	**P value**
24-h HR, bpm	41/46 (89%)	32/46 (70%)	19/92 (21%)	−1.0 (−14.9 to 12.8)	0.88
Night HR, bpm	27/46 (59%)	22/46 (48%)	43/92 (47%)	−2.1 (−17.3 to 13.0)	0.78

Supplementary Table S3: Multiple-imputation sensitivity analysis of longitudinal heart-rate parameters. Negative estimates indicate a greater reduction (or smaller increase) from baseline to 12 months in the ertugliflozin group relative to placebo. Multiple imputation was performed as a sensitivity analysis and does not eliminate potential bias arising from structurally unavailable measurements.

Manufacturer	**Group (0=placebo, 1=Ertugliflozin)**	**Device n**	**24h-HR V1**	**24h-HR V2**	**Night HR V1**	**Night HR V2**	**HRV V1**	**HRV V2**	**Intrathoracic mpedance V1**	**Intrathoracic impedance V2**	**Activity V1**	**Activity V2**
Boston Scientific	**0**	**11**	**10**	**7**	**3**	**2**	**5**	**5**	**2**	**2**	**9**	**7**
	**1**	**7**	**6**	**6**	**2**	**3**	**2**	**2**	**1**	**1**	**6**	**5**
Medtronic	**0**	**8**	**6**	**4**	**5**	**4**	**6**	**4**	**1**	**3**	**7**	**8**
	**1**	**6**	**6**	**2**	**5**	**2**	**5**	**4**	**2**	**1**	**6**	**4**
Abbott	**0**	**5**	**4**	**5**	**5**	**4**	**0**	**0**	**1vRe**	**1**	**3**	**4**
	**1**	**7**	**7**	**6**	**5**	**5**	**2**	**1**	**3**	**4**	**7**	**6**
Biotronik	**0**	**0**	**0**	**0**	**0**	**0**	**0**	**0**	**0**	**0**	**0**	**0**
	**1**	**2**	**2**	**2**	**2**	**2**	**2**	**1**	**1**	**1**	**2**	**2**
Total	**0**	**24**	**20**	**16**	**13**	**10**	**11**	**9**	**4**	**6**	**19**	**19**
	**1**	**22**	**21**	**16**	**14**	**12**	**11**	**8**	**7**	**7**	**21**	**17**
Overall	**Total**	**46**	**41**	**32**	**27**	**22**	**22**	**17**	**11**	**13**	**40**	**36**
Availability			**89%**	**70%**	**59%**	**48%**	**48%**	**37%**	**24%**	**28%**	**87%**	**78%**

Supplementary Table S4: Manufacturer-specific availability of device-derived remote-monitoring parameters. Numbers indicate participants with an available measurement for the respective parameter and time point. V1 represents baseline and V2 the 12-month follow-up. Device-derived parameters should not necessarily be considered measurement-equivalent across manufacturers because sensing methods, signal processing, calculation algorithms, and reporting conventions differ between platforms. HR, heart rate; HRV, heart rate variability.
